# Goal-directed osteoporosis treatment: ASBMR/BHOF task force position statement 2024

**DOI:** 10.1093/jbmr/zjae119

**Published:** 2024-07-29

**Authors:** Felicia Cosman, E Michael Lewiecki, Richard Eastell, Peter R Ebeling, Suzanne Jan De Beur, Bente Langdahl, Yumie Rhee, Ghada El-Hajj Fuleihan, Douglas P Kiel, John T Schousboe, Joao Lindolfo Borges, Angela M Cheung, Adolfo Diez-Perez, Peyman Hadji, Sakae Tanaka, Friederike Thomasius, Weibo Xia, Steven R Cummings

**Affiliations:** Department of Medicine, Columbia University College of Physicians and Surgeons, New York*,* NY 10032, United States; New Mexico Clinical Research & Osteoporosis Center, Division of Metabolic Bone Diseases, Albuquerque, NM 87106, United States; Division of Clinical Medicine, University of Sheffield, Sheffield S10 2RX, United Kingdom; Department of Medicine, School of Clinical Sciences, Monash University, Clayton, VIC 3168, Australia; Division of Endocrinology and Metabolism, University of Virginia, Charlottesville, VA 22908, United States; Department of Endocrinology, Aarhus University Hospital, Aarhus N 8200, Denmark; Department of Clinical Medicine, Aarhus University, Aarhus N 8200, Denmark; Department of Internal Medicine, Severance Hospital, Endocrine Research Institute, Yonsei University College of Medicine, Seoul 03722, South Korea; Calcium Metabolism and Osteoporosis Program, WHO Collaborating Center for Metabolic Bone Disorders, American University of Beirut, Beirut 1107, Lebanon; Marcus Institute for Aging Research, Hebrew Senior Life, Boston, MA 02131, United States; Department of Medicine, Beth Israel Deaconess Medical Center, Harvard Medical School, Boston, MA 02215, United States; Division of Research and Evaluation, HealthPartners Institute, Bloomington MN 55425, United States; Division of Health Policy and Management, University of Minnesota, Minneapolis, MN 55425, United States; Brazil Clinical Research Center, Brasilia 71625-175, Brazil; Department of Medicine and Joint Department of Medical Imaging, University Health Network, Toronto, ON M5G 2C4, Canada; Centre of Excellence in Skeletal Health Assessment, University of Toronto, Toronto, ON M5G 2C4, Canada; Department of Medicine, Institute Hospital del Mar of Medical Investigation, Barcelona 08003, Spain; Department of Obstetrics and Gynecology, Frankfurt Center of Bone Health and Phillipps-University of Marburg, Frankfurt 60313, Germany; Department of Orthopaedic Surgery, Faculty of Medicine, The University of Tokyo, Hongo, Bunkyo-ku, Tokyo 113-0033, Japan; Department of Clinical Osteology, Frankfurt Center of Bone Health and Endocrinology, Frankfurt 60313, Germany; Department of Endocrinology, Peking Union Medical College Hospital, Chinese Academy of Medical Sciences & Peking Union Medical College, Beijing 100730, China; San Francisco Coordinating Center, CPMC Research Institute, Department of Epidemiology and Biostatistics, University of California, San Francisco, CA 94158, United States

**Keywords:** practice/policy-related issues, anabolics, therapeutics, antiresorptives, therapeutics, osteoporosis, diseases and disorders of/related to bone, DXA, analysis/quantitation of bone

## Abstract

The overarching goal of osteoporosis management is to prevent fractures. A goal-directed approach to long-term management of fracture risk helps ensure that the most appropriate initial treatment and treatment sequence is selected for individual patients. Goal-directed treatment decisions require assessment of clinical fracture history, vertebral fracture identification (using vertebral imaging as appropriate), measurement of bone mineral density (BMD), and consideration of other major clinical risk factors. Treatment targets should be tailored to each patient’s individual risk profile and based on the specific indication for beginning treatment, including recency, site, number and severity of prior fractures, and BMD levels at the total hip, femoral neck, and lumbar spine. Instead of first-line bisphosphonate treatment for all patients, selection of initial treatment should focus on reducing fracture risk rapidly for patients at very high and imminent risk, such as in those with recent fractures. Initial treatment selection should also consider the probability that a BMD treatment target can be attained within a reasonable period of time and the differential magnitude of fracture risk reduction and BMD impact with osteoanabolic versus antiresorptive therapy. This position statement of the ASBMR/BHOF Task Force on Goal-Directed Osteoporosis Treatment provides an overall summary of the major clinical recommendations about treatment targets and strategies to achieve those targets based on the best evidence available, derived primarily from studies in older postmenopausal women of European ancestry.

## Introduction

Since our last report in 2017,^1^ the emergence of new medical evidence and development of new therapeutic agents have prompted a need to revisit the concept of goal-directed treatment (treat-to-target) for osteoporosis. For these reasons, the American Society for Bone and Mineral Research (ASBMR) and Bone Health & Osteoporosis Foundation (BHOF, formerly National Osteoporosis Foundation) re-assembled an international task force of clinical scientists and expert physicians to update the previous report and develop a pragmatic approach to aid clinicians in managing patients with osteoporosis. Multiple drafts of this document were extensively vetted by all authors. Comments, suggestions and edits were carefully considered and text was accepted or modified until all authors were in agreement. Pharmaceutical companies provided no funding for the development of this manuscript and had no role in its concept, writing, or revisions. There was no external funding from any source. All coauthors approved the final version of the position statement.

The most recent guidelines from the American Association of Clinical Endocrinology, Endocrine Society, North American Menopause Society (now The Menopause Society), BHOF, European Society for Clinical, and Economic Aspects of Osteoporosis and Osteoarthritis/International Osteoporosis Foundation and National Osteoporosis Guideline Group^2–7^ have included a new very high fracture risk category. A subset of very high-risk patients is also at imminent risk of fracture for 2 yr following an incident fracture^2^^,^^4^^,^^5^^,^^8–16^ or after multiple prior fractures.^2^^,^^17–20^

Since the last report, two new osteoanabolic agents, abaloparatide and romosozumab, have been approved for treatment of patients with osteoporosis in the USA and in many other countries. Head-to-head studies in high or very high-risk patients demonstrate that osteoanabolic agents are more effective in preventing osteoporotic fractures than bisphosphonates^21–25^ and denosumab.^26^ Additionally, the Foundation for the National Institutes of Health/Study to Advance bone mineral density (BMD) as a Regulatory Endpoint collaboration (FNIH/SABRE) showed a strong relationship between BMD increases with treatment compared with placebo, and the magnitude of anti-fracture efficacy in a meta-regression of over 50 clinical trials.^27–29^ In clinical trials, the increase in total hip (TH) and femoral neck (FN) BMD in treated patients compared with placebo explained a somewhat greater proportion of the anti-fracture effect than change in lumbar spine (LS) BMD.^28^

Data from trials with antiresorptive and osteoanabolic agents show an inverse relationship between the BMD level achieved in patients on treatment and the subsequent risk of fracture.^30–33^ New evidence shows that TH BMD appears to be the most useful treatment target because it consistently predicts the risk of both vertebral and nonvertebral fractures, whereas the LS BMD level after treatment predicts the risk of vertebral fracture but does not predict the risk of nonvertebral fractures as consistently.^33^ In addition, new evidence provides guidance for identifying a BMD target.^32^^,^^33^ Other studies have confirmed the benefit of treating with an osteoanabolic medication before an antiresorptive drug, compared with the reverse order, to maximize gains in BMD, particularly in the hip^34–38^ and the probability of achieving different BMD treatment targets beginning with osteoanabolic rather than antiresorptive agents at varying starting BMD levels.^39–41^

It is common clinical practice and a requirement of many health insurers to prescribe an oral bisphosphonate as initial treatment for all patients with osteoporosis, unless a contraindication is present. However, this “step therapy” approach does not provide the most effective treatment for all high and very high-risk patients. In contrast, goal-directed treatment individualizes treatment decisions based on an individual’s fracture risk, taking into account fracture history, BMD, and other risk factors, aiming at prespecified individualized treatment targets. Based on these considerations, some patients will benefit most from osteoanabolic therapy as initial treatment.

Goal-directed therapy is a strategy for the long-term management of patients receiving treatment for osteoporosis. Achieving treatment targets might require intensification of therapy if a fracture occurs or the patient remains far from a BMD target despite osteoporosis treatment. This intensification could include replacing a bisphosphonate with denosumab, replacing a bisphosphonate or perhaps denosumab with an osteoanabolic agent, or adding an osteoanabolic agent to ongoing treatment with a bisphosphonate or denosumab. It might also include a repeat course of osteoanabolic medication. It must be acknowledged, however, that the BMD effects of switching from antiresorptive to osteoanabolic agents are not as robust as those seen when initiating treatment with an osteoanabolic agent (especially when switching from denosumab).^38^ In addition, the evidence supporting the safety and efficacy of repeat courses of osteoanabolic agents is extremely limited. Extended use beyond 24 mo and repeated use of branded teriparatide is approved in the USA when a patient remains at or has returned to having a high risk for fracture.

This report represents the consensus of the ASBMR/BHOF Task Force on Goal-Directed Osteoporosis Treatment, based on interpretation of the best evidence available. This position statement is not a clinical guideline. It begins with a summary overview of the main principles, followed by a description of the evidence underlying these principles. It is focused on postmenopausal White women for whom the vast majority of evidence is available. For physicians who are not confident prescribing goal-directed therapy or managing very-high risk patients, referral to an osteoporosis specialist should be considered.

### Summary of major principles

#### Treatment targets

Treatment targets should be individualized, based in large part on the specific indication for beginning treatment, including recency of fracture, number and site of prior fractures, severity of vertebral fracture(s), BMD at the TH, FN, and/or LS, age, and other strong risk factors for fracture.

- *Patients at imminent risk of fracture:* Patients with a recent fracture are at very high risk of more fractures over the next 2 yr; this risk is largely independent of baseline T-score. The treatment goal for these patients is to rapidly and maximally reduce fracture risk. Greater BMD increases are associated with greater reduction in fracture risk. Some patients who have had multiple prior fractures (even if not within the last 2 yr) are also at imminent risk. Many of these patients also have sustained very-high risk. For these patients, the treatment goal is rapid and maximal reduction in fracture risk, but more sustained treatment is likely required than for a single recent fracture.
- *For patients who are not at imminent risk of fracture, baseline T-scores, fracture history, and other major risk factors guide the selection of treatment targets.*
- *Patients with baseline T-score ≤ −2.5:* For patients with baseline T-scores *<* −2.5 at the TH, FN, and/or LS, the treatment target is a T-score level at least > −2.5 at the respective skeletal sites.
  - In patients on osteoporosis treatment, TH T-score best reflects subsequent fracture risk at both vertebral and nonvertebral sites. In patients on osteoporosis treatment, T-score at the FN also reflects subsequent risk of fracture at both vertebral and nonvertebral sites. Since reproducibility is better for the TH than the FN, it is the preferred region of interest for a treatment target. For patients with an isolated T-score *<* −2.5 at the FN, the FN T-score can be the treatment target. Improving T-score to levels > −2.5 is associated with lower risk for nonvertebral and vertebral fractures. A T-score > −2.5 should be the minimum treatment target. In some patients, achieving a higher T-score target might be warranted, based on other risk factors.
  - In patients on osteoporosis treatment, LS T-score reflects subsequent risk of vertebral fracture, but the association with nonvertebral fracture is less consistent. Improving LS T-score to levels > −2.5 is associated with lower vertebral fracture risk; a T-score > −2.5 should be the minimum treatment target. In some patients, achieving a higher target T-score might be warranted based on other risk factors.
  - For countries with different T-score intervention thresholds, T-score targets should be adjusted accordingly.
- *Patients with baseline T-score >* −*2.5:* For patients with baseline T-scores > −2.5 who are recommended for treatment because of high fracture risk (such as prior fracture or high-risk medical conditions), increasing BMD remains associated with reduced fracture risk. Greater BMD increases with larger T-score improvements are associated with greater fracture-risk reduction, but a T-score target is not easily defined in these patients.

#### Selecting treatment to achieve treatment targets

- *Initial treatment:* Selection of initial treatment should consider the probability that a treatment target can be attained over a reasonable period of time, with greater urgency for patients at imminent fracture risk (recent fracture or some multiple prior fractures). Data to guide these decisions include the likelihood that a treatment can provide at least a 50% probability of attaining the T-score target over 3 yr, depending on the initial BMD. For some patients, it might be appropriate to select treatment to achieve a higher T-score target, reach the treatment target faster, or provide a higher probability of achieving the treatment target.
- *Sequence of treatment:* Treatment sequence is important for attaining T-score targets particularly in patients with baseline T-scores far below the treatment target. Selection of initial therapy should acknowledge the differential impact on BMD for osteoanabolic-first versus antiresorptive-first treatment sequences. Greater and faster BMD increases at the hip and spine are seen with osteoanabolic-first followed by antiresorptive treatment strategies, compared with the reverse order.

#### Determining if treatment targets have been achieved

To determine whether a treatment target has been achieved requires repeat BMD testing and assessment for new fractures, including vertebral imaging. Having a baseline vertebral image before starting treatment allows confirmation that an incident vertebral fracture has occurred on follow-up vertebral imaging. If a patient experiences one or more new fractures, it indicates that the most important treatment target has not been met, regardless of the T-scores achieved. Monitoring for achievement of a treatment target is distinct from monitoring for treatment responses. To achieve a treatment target, a treatment response is necessary. Treatment response, which can be assessed by measurement of biochemical turnover markers, depends on patient adherence and type and duration of prior osteoporosis medication.

- When a treatment target has not been achieved or is unlikely to be achieved, consider changing to more potent therapy (or continuing the highest potency treatment sequence).
- When a treatment target has been reached, maintenance of BMD is the focus. This may involve continuing treatment, changing treatment, pausing treatment, or administering intermittent bisphosphonates.

### Review of the evidence to support major principles and actionable steps in patient management

To establish a treatment target and select initial therapy requires assessment of fracture risk, including vertebral imaging when appropriate, and BMD measurement when available. Indications for BMD testing and vertebral imaging are available in the BHOF Clinician’s Guide^6^ and the International Society for Clinical Densitometry Position Statements.^42^ Treatment targets should reflect the underlying clinical scenarios; specific BMD treatment targets and choice of therapeutic agents should be individualized.

### Treatment targets and selection of treatment for patients at imminent risk

#### Recent fracture and imminent risk of subsequent fracture

**Figure 1:**
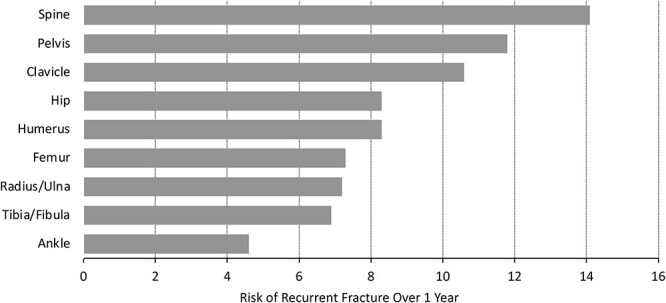
One-year risk of recurrent fracture in women *>* 65 years of age, based on site of initial fracture, from Medicare database of 377,561 women with first fracture. Adapted from reference 8.

Multiple recent studies in different regions of the world confirm the importance of recent fracture as a predictor of subsequent fracture.^8–16^ Overall, these large database studies show that the risk of fracture is increased dramatically for up to 2 yr after the occurrence of the first fracture, with the annual risk remaining high but declining after that time. Risk increases very rapidly after an initial fracture, with the highest risk seen within the first few months after the fracture.^10^ One of the most comprehensive studies utilized a Medicare database to evaluate 377,500 women age 65 yr and older who had a first clinical fracture.^8^ Women with fractures of the digits, skull, patella, sternum, scapula, and ribs were excluded. Fracture risk was assessed for up to 5 subsequent yr. Average risk of subsequent fracture was 10% in the very next year and 18% in the 2 yr following the first fracture. The site of the first fracture was associated with varying risks of subsequent fracture (Figure 1).^8^ The magnified short-term risk was particularly high after vertebral and pelvic fractures and lowest for ankle fractures, though even ankle fractures were still associated with a subsequent 1-yr risk of just below 5% and 2-yr risk of almost 10% (which is still considered imminent risk).^43^ Therefore, in patients at imminent risk, especially those with recent fractures of the spine, hip, and pelvis, rapid and maximum fracture risk reduction is the first and most important treatment target.

#### Multiple prior fractures and imminent risk of subsequent fracture

Patients with multiple prior fractures may also be at imminent risk for more fractures.^2^^,^^17–20^ Fracture site, severity, and time from last fracture occurrence remain important determinants of subsequent risk. In many patients with two or more fractures, rapid and maximum fracture risk reduction is the most important treatment goal. This is particularly pertinent for women with fracture sites that include spine, hip, or pelvis and for patients whose fractures were not remote (more than 10 yr earlier). Additionally, in many patients with multiple fractures, very-high risk persists, especially if there are no modifiable risk factors. Treatment targets might include attaining higher T-score targets than specified below. Furthermore, these patients might require a more prolonged and intensive course of therapy even after achieving T-score targets.

#### Selecting initial treatment for patients at imminent risk

Since osteoanabolic agents reduce fracture risk faster and to a greater extent than antiresorptive agents,^21–26^ osteoanabolic therapy may be better for patients at imminent fracture risk, especially after recent major fractures such as spine, hip, and pelvis (Figure 1).^8^ Initiating treatment with osteoanabolic agents followed by antiresorptive agents also increases BMD more than the reverse treatment sequence. Therefore, BMD treatment targets are more likely to be achieved rapidly with an osteoanabolic-first treatment sequence. For patients with other recent fractures, osteoanabolic agents, bisphosphonates, or denosumab may be appropriate, depending site of fracture and BMD. A consensus regarding which recent non-hip, non-spine, non-pelvis fractures should prompt use of osteoanabolic therapy versus bisphosphonates or denosumab is needed.

### Treatment targets and selection of treatment for patients with T-scores ≤ −2.5

#### Why is BMD a good target for osteoporosis treatment and what is the best skeletal site?

In a large meta-regression of pharmaceutical clinical trials, 2-yr mean BMD differences between active and placebo-treated patients in the TH, FN, and LS were associated with fracture risk reduction;^27–29^ larger mean BMD increases predicted greater reductions in fracture risk. Mean TH and FN BMD increments explained a somewhat larger proportion of fracture risk reduction than mean LS BMD increments (Table 1).^28^ LS BMD is more likely affected by degenerative artifact and aortic calcification. Of the two hip regions of interest, the TH has better reproducibility than the FN.^28^

**Table 1:** Proportion of treatment-related fracture reduction effect explained by BMD increment at the TH, FN, and LS (95% CI).

	Total Hip	Femoral Neck	Lumbar Spine
**Vertebral fracture**	59% (50-69)	61% (51-72)	31% (19-44)
**Nonvertebral fracture**	63% (38-88)	67% (40-95)	52% (23-82)
**Hip fracture**	48% (21-76)	44% (12-77)	42% (9-75)

Adapted from reference 28.

**Figure 2:**
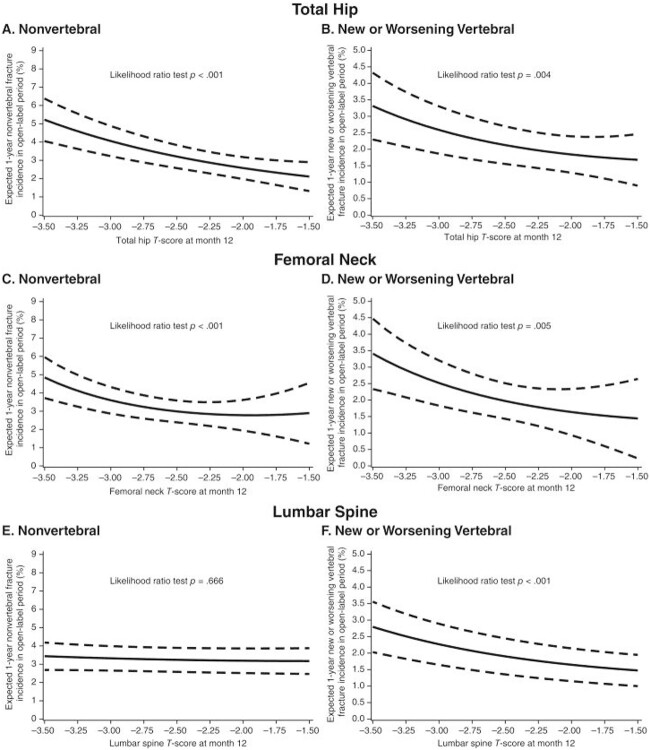
Relationship between T-score attained after one year of treatment with either alendronate or romosozumab and subsequent risk of nonvertebral (left) or vertebral fracture (right) for the total hip (A and B), femoral neck (C and D) and lumbar spine (E and F).^33^

Mean TH BMD level after treatment is predictive of subsequent risk of both nonvertebral and vertebral fractures.^30–33^ The relationship between mean LS BMD level after treatment has not been tested in as many clinical trials, since several studies did not assess LS BMD in all individuals at all time points.^30^^,^^31^ In trials where LS BMD has been measured in the full population, mean LS T-score reflects subsequent risk of vertebral fracture consistently; however, the association with nonvertebral fracture is not universal. For example, in a post hoc analysis from the ARCH study (Active-controlled fracture study in postmenopausal women with osteoporosis at high risk), mean LS BMD attained after 1 yr of treatment with alendronate or romosozumab was associated with subsequent risk of vertebral fracture but not nonvertebral fracture (Figure 2).^33^

For all of these reasons, the single best skeletal site for a T-score target is the TH. This might be important in some clinical situations, for example where the TH T-score is already > −2.5, but LS T-score is below. Medication choice might differ in this type of patient, rather than the reverse situation where LS T-score is > −2.5, but TH T-score is lower (see below). In patients who have only a FN T-score *<* −2.5 or a LS T-score *<* −2.5, a T-score > −2.5 at the respective skeletal site should be the treatment target.

#### What is the rationale for choosing the minimum target of > −2.5?

In some countries, a T-score *<* −2.5 represents an indication for pharmacologic treatment regardless of other risk factors.^6^ However, since fracture risk is dependent on other factors, notably age and prevalent fracture,^44^^,^^45^ a T-score > −2.5 should be considered the *minimal target*. For countries with different or no T-score intervention thresholds, T-score targets should be adjusted accordingly.^46^

**Figure 3:**
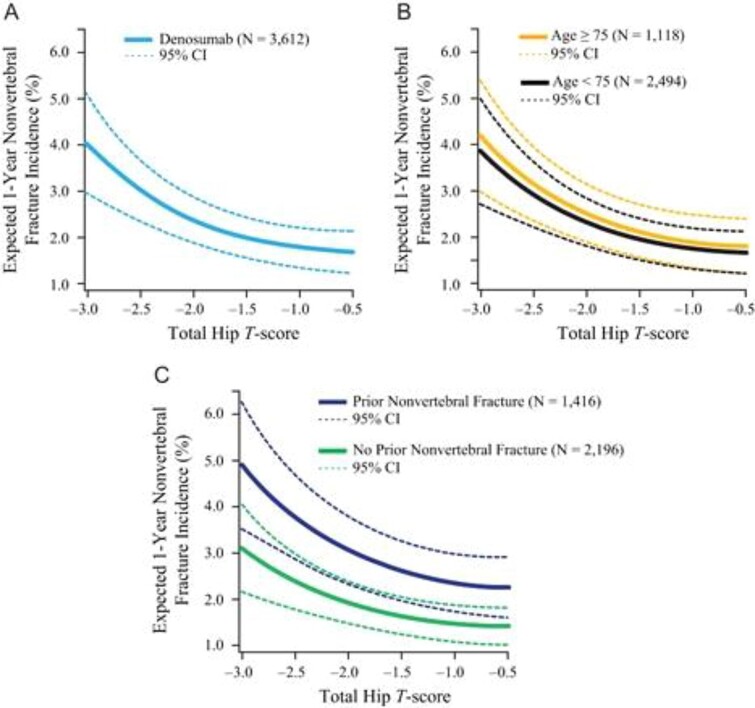
Relationship between T-score attained on denosumab and subsequent risk of nonvertebral fracture in full population (A), by age < 75 vs. *>* 75 yr (B), and by prior history of fracture (C).^32^

Patients with other important risk factors might warrant higher T-score targets. This is demonstrated very clearly in analyses from the denosumab FREEDOM trial (Fracture Reduction Evaluation of Denosumab in Osteoporosis Every 6 Mo) and its extension.^32^ The relationship between attained T-score on denosumab treatment and subsequent fracture risk is maintained in individuals with and without prior fracture, but the risk at all BMD levels is much higher in those with a fracture history (Figure 3).^32^ These data support setting higher T-score targets in patients with a history of fracture. Higher T-score targets might also be suggested for patients with advanced age, recent falls history, and poor physical function.^47–49^ In these patients, T-score targets of −2.0 or even −1.5 might be appropriate. In the ARCH trial, where all patients had fractures at entry, progressive lowering of subsequent fracture risk was observed as 1 yr TH T-scores approached −1.5.^33^

A higher T-score target might also be considered in patients for whom a pause in medication is being considered. Anyone who stops osteoporosis medication will lose bone eventually, though the rate of loss is dependent on what medication is being discontinued. Bone loss rates after stopping bisphosphonates are much slower than bone loss rates after stopping all other agents. Therefore, medication pauses should only be considered in patients whose last treatment is a bisphosphonate. Aiming for a higher T-score in those patients being considered for a medication pause might permit maintenance of T-score above −2.5 even after the expected bone loss occurs (see section below on BMD maintenance for further discussion).

#### Selecting initial treatment to achieve target BMD

The choice of therapeutic agent is dependent on which skeletal site has the lowest T-score and exactly what the starting BMD is at that site. When selecting initial treatment, the clinician should consider the probability that BMD targets can be attained with a specific treatment over a reasonable period of time (see “Probability of Attaining BMD Treatment Targets” below). Selection of initial therapy should also acknowledge the differential impact on BMD for osteoanabolic-first versus antiresorptive-first treatment sequences and the differential effects of medications on hip and spine BMD.

Goal-directed therapy choices should also consider other important clinical factors:

- *In patients with TH T-score −2.5 to −2.8 (inclusive) and LS T-score −2.5 to −3.0 (inclusive)*: bisphosphonates, denosumab, and osteoanabolic agents are all likely to enable the achievement of T-scores > −2.5 at both TH and LS within 3 yr. Treatment choices should consider a patient’s fracture history, including the skeletal site and timing of prior fracture. For patients with no prior fracture or a fracture other than spine, hip, or pelvis, bisphosphonates and denosumab are both highly likely to enable achievement of treatment targets within 3 yr and are the most appropriate choice. In contrast, in patients who have had a major fracture such as the spine, hip, or pelvis, osteoanabolic agents should be considered. This is most important if those fractures occurred somewhat recently (eg, more than 2 yr before, but within the last decade). In those patients, choosing osteoanabolic agents as initial therapy might enable patients to achieve treatment targets faster, to achieve higher T-score targets, or to have a higher probability of achieving the treatment target when beginning treatment.^39–41^ Individual patient factors and local/regional guidance should be considered when deciding treatment based on site of prior fracture or T-score intervention threshold.^46^
- *In patients with very low BMD (TH T-scores < −2.8 or LS T-scores < −3.0):* osteoanabolic agents might be required to achieve BMD treatment targets (see next section on probability of attaining BMD targets). Osteoanabolic agents and osteoanabolic-first treatment sequences produce larger BMD gains in both TH and LS compared with antiresorptive agents alone. Therefore, osteoanabolic agents should be considered as initial therapy for these patients when possible. In patients who start with a TH T-score *<* −3.5, it might be impossible to achieve a treatment target with any current medication in 3 yr; however, treatment decisions should aim to improve TH T-score to levels as close to the target as possible. Bisphosphonates or denosumab might be the first choice in some patients with no other risk factors, especially in countries where T-score intervention thresholds are lower.

#### Probability of attaining BMD treatment targets

Table 2 provides estimates of the lowest starting TH or LS T-score that would allow at least a 50% probability of attaining a T-score > −2.5 at that site, based on data available for different therapeutic agents over ~3 yr of treatment. These data can be helpful in making initial treatment decisions.

**Table 2:** Lowest baseline T-score that permits *>* 50% of women to achieve a T-score > −2.5 in approximately 3 yr.

	Total Hip	Lumbar Spine
**Alendronate**	−2.7	−3.0
**Denosumab**	−2.8	−3.1
**Romosozumab/Alendronate**	−2.9	−3.5
**Abaloparatide/Alendronate**	−2.9	−3.5
**Romosozumab/Denosumab**	−3.1	−3.7

Adapted from references 39–41.

#### Probabilities with romosozumab followed by alendronate or denosumab versus alendronate only

**Figure 4:**
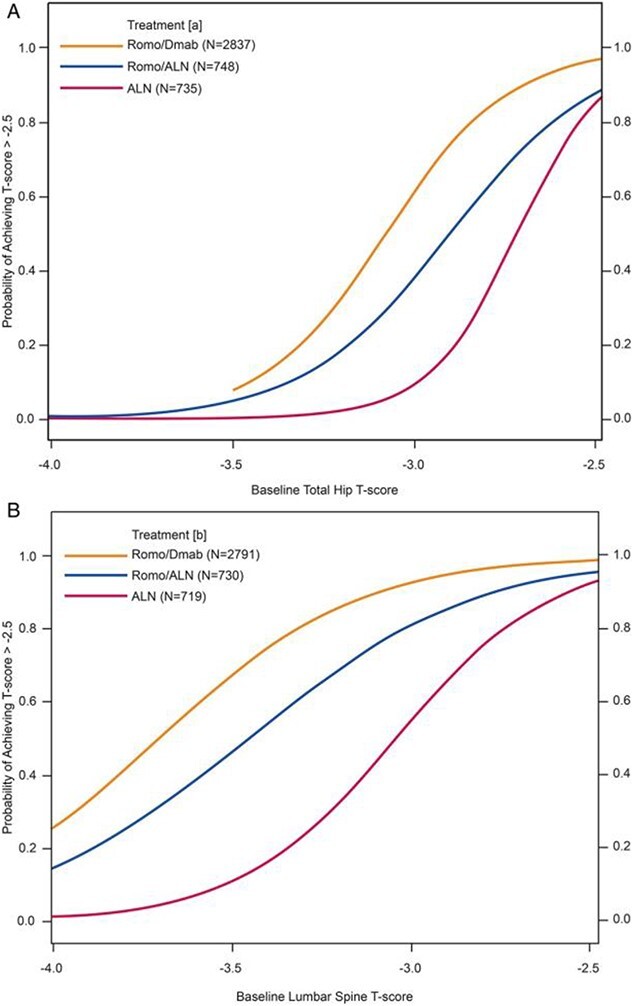
Probability of attaining T-score above −2.5 for TH (A) and LS (B) over 3 yr during treatment with alendronate, romosozumab followed by alendronate, or romosozumab followed by denosumab, based on starting T-score.^39^

In a post hoc analysis of the ARCH and FRAME (Fracture study in postmenopausal women with osteoporosis) studies, the probability of attaining BMD targets over a total of 3 yr of therapy with alendronate alone, 1 yr of romosozumab followed by alendronate, or 1 yr of romosozumab followed by denosumab was determined (Figure 4; Table 2).^39^ In patients with TH T-score of −2.7, the probability of achieving a TH T-score > −2.5 with alendronate alone over 3 yr was > 50%, whereas this probability was only 9% for those who started with TH T-score of −3.0. For romosozumab followed by alendronate, the corresponding probabilities were 73% and 38%, respectively. For romosozumab followed by denosumab, these probabilities were 90% and 61%, respectively.^39^

For women who began with LS T-scores of −3.0, the probability of achieving a LS T-score above −2.5 with alendronate alone was 55%, while it was only 11% with a starting LS T-score of −3.5. With romosozumab followed by alendronate, these probabilities were 81% and 46% respectively for starting T-scores of −3.0 and −3.5, respectively. With romosozumab followed by denosumab, the probabilities were 93% and 67%, respectively.

#### Probabilities with abaloparatide for 18 mo followed by alendronate for 2 yr

In a post hoc analysis of the ACTIVE study (Abaloparatide Comparator Trial in Vertebral Endpoints) and its extension, the probability of attaining BMD targets with abaloparatide for 18 mo followed by alendronate for 2 yr was determined.^40^ Women with baseline TH T-scores as low as −2.9 and baseline LS T-scores as low as −3.5 had a > 50% probability of attaining a T-score above −2.5 at each respective site with abaloparatide followed by alendronate (Table 2).

#### Probabilities with denosumab over 3 yr

In a post hoc analysis of the FREEDOM study, the probability of attaining BMD targets with 3 yr of denosumab treatment was determined.^41^ There was a > 50% probability that women with starting TH T-scores *>* −2.8 and LS T-scores *>* −3.1 could achieve target T-scores above −2.5 with 3 yr of denosumab treatment (Table 2).

**Table 3:** Relationship between % BMD increase and T-score change in the TH and LS at 1 yr after treatment with romosozumab or alendronate.

	Romosozumab (*n* = 1739)		Alendronate (*n* = 1726)	
	Mean % change BMD	Mean change T-score	Mean % change BMD	Mean change T-score
**Total hip**	6.3%	0.31	2.9%	0.15
**Lumbar spine**	13.9%	0.90	5.1%	0.34

Adapted from reference 33.

### Importance of treatment sequence

BMD increases with osteoanabolic agents are lower, particularly in the TH, when patients transition from a bisphosphonate or denosumab. Upon transition from a bisphosphonate to teriparatide, TH BMD declines consistently for at least 12 mo and hip strength is not improved for at least 1 yr.^34^^,^^35^ Data are not currently available to assess the BMD effects of a treatment sequence beginning with an antiresorptive and switching to abaloparatide. When patients on denosumab are switched to teriparatide, TH BMD declines rapidly and remains below the on-denosumab baseline for a full 2 yr.^36^ Upon transition from a bisphosphonate to romosozumab, TH BMD and hip strength improve^35^ although not as markedly as seen with romosozumab in treatment naïve individuals. After 1 yr of denosumab treatment, upon transition to romosozumab, TH BMD remains stable;^50^ however, the BMD effect with romosozumab might differ after a longer preceding denosumab treatment duration. LS BMD increments are also lower with osteoanabolic agents upon transition from antiresorptive agents; however, the difference is not as marked as it is in the TH.^34–38^^,^^50^

#### Choice of treatment after osteoanabolic

In patients initially treated with osteoanabolic therapy for 1-2 yr (as appropriate for the agent used), the subsequent choice of antiresorptive agent should take into account the repeat BMD level and other clinical risk factors. Patients who are still far from BMD targets might benefit from denosumab to attain BMD targets, since denosumab increases BMD more than bisphosphonates after osteoanabolic treatment.^38^ In patients who are close to BMD targets after osteoanabolic treatment, intravenous or oral bisphosphonates can be considered as follow-up therapy. In some countries, it might be possible to consider another course of osteoanabolic therapy some years later for those who still have T-scores below −2.5. If another course of osteoanabolic therapy is foreseen, bisphosphonates may be the better intermediate treatment choice. This would avoid the overshoot bone remodeling seen after denosumab discontinuation, which might mitigate the effect of the second course of osteoanabolic treatment.^38^

### Treatment targets and selection of treatment for patients with T-scores > −2.5

More than half of all patients who have adulthood fractures have BMD levels above osteoporosis range.^51–53^ For patients with baseline T-scores > −2.5 who have had a single prior fracture that occurred more than 2 yr earlier, subsequent risk might differ substantially by skeletal site and time since fracture. Prior vertebral, hip, and pelvic fractures are associated with higher and more persistent risk than other fractures. For higher-risk patients who require treatment, increasing BMD is associated with reduced fracture risk.^54^ There is a paucity of evidence to guide the actual BMD level to target in these patients. Percentage increase in BMD is a function of baseline BMD, with lower percentage increases expected in those with higher baseline BMD.^54^ Another level of uncertainty is how percentage changes in BMD relate to T-score unit changes. These relationships are specific for different populations at different starting BMD levels. For example, in the ARCH study, where all women began with T-scores *<* −2.0, mean BMD percentage increments with 1 yr of alendronate or romosozumab treatment were compared with mean T-score changes (Table 3).^33^ With 1 yr of alendronate, BMD increments of 2.9% in TH and 5.1% in LS corresponded to T-score unit changes of 0.2 and 0.3, respectively. With romosozumab, BMD increases of 6% in TH and 14% in LS BMD were associated with T-score unit increases of 0.3 and 0.9. BMD gain and T-scores continue to increase over at least 1-2 subsequent yr. TH T-score increments of at least 0.2 units and LS increments of at least 0.5 units are achievable over 3 yr with most therapies (bisphosphonates, denosumab, and osteoanabolic agents) and would be expected to reduce risk of both vertebral and nonvertebral fracture.^55–62^ Therefore, these T-score changes can be considered treatment targets in these patients.

For patients with no prior fracture, T-score above −2.5, but high fracture probability according to a fracture risk algorithm, initiation of treatment with an antiresorptive agent may be appropriate, with a goal of increasing TH T-score by 0.2 (3%) and LS T-score by 0.5 (6%).

**Figure 5:**
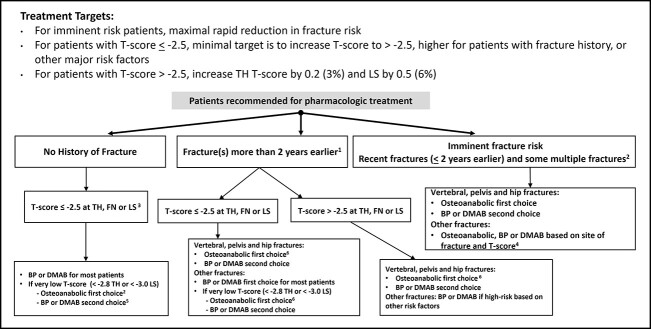
Goal directed therapy algorithm. Treatment targets and initial treatment selection, guided by fracture history (site, number and recency) and BMD. ^1^Risk likely differs based on fractures that occurred a few years earlier vs. very remote events (eg 15 yr earlier). ^2^Many, but not all, patients with multiple fractures are at imminent risk, based on fracture sites and time from fracture occurrence. ^3^In some countries, T-score intervention thresholds are lower; T-score targets should be adjusted accordingly. ^4^There is no consensus about which recent fracture sites should be recommended for osteoanabolic treatment vs. BP or DMAB or what T-score should prompt use of osteoanabolic treatment in patients with recent “other fractures.” ^5^BP or DMAB might be first choice in some patients with no other risk factors, especially in countries where T-score intervention thresholds are lower. ^6^Choosing osteoanabolic agents provides a higher probability of achieving treatment targets, achieving treatment targets faster, and achieving higher T-score targets. Abbreviations: TH, total hip; FN, femoral neck; LS, lumbar spine; BP, bisphosphonates; DMAB, denosumab.

An algorithm, which presents treatment targets and principles guiding selection of initial treatments for individual patients, is provided in Figure 5.

### Determining if treatment targets have been achieved

Determining whether patients have achieved treatment targets is distinct from monitoring for treatment responses; patients might have had a good treatment response yet still be at high risk of fracture if treatment targets have not been achieved.

#### When treatment targets have not been met

Patients with clinical fractures or confirmed vertebral fractures while on medication have not achieved treatment targets and are at imminent risk of more fractures. While a fracture on treatment does not necessarily represent treatment failure, it does suggest that fracture risk is higher than previously recognized. After an evaluation for secondary causes of osteoporosis, interventions to reduce falls risk and improve treatment adherence should be implemented and patients should be started or continued on the most potent medication (or medication sequence) for at least the next 2 yr. This strategy could also consider switching to or adding an osteoanabolic agent to ongoing antiresorptive medication. In women who have already been treated with osteoanabolic medication and remain at very high risk, another course of osteoanabolic medication (either using the same or different medication) could be considered. The optimal timing of a second course of treatment and how to maximize BMD gain after antiresorptive therapy is unknown, although repeated use of branded teriparatide is approved in some countries, if a patient remains at or has returned to a high risk for fracture. This approach might also be appropriate for women whose BMD levels remain below the target or those who lose BMD despite therapy. Continuing an antiresorptive medication, such as denosumab, is also a potential strategy, especially when osteoanabolic options are limited.^63^

#### When treatment targets have been achieved

For patients who have achieved treatment targets, benefits are expected to wane if medication is discontinued. BMD declines rapidly after discontinuation of most agents and important clinical consequences might occur, such as multiple vertebral fractures after denosumab discontinuation.^64^^,^^65^ Upon cessation of denosumab, after short-term use (< 3 yr), transition to bisphosphonates appears to be effective at mitigating bone loss and risk of multiple vertebral fractures, but the most effective regimen after long-term use remains uncertain.^38^^,^^64^^,^^65^ In contrast to denosumab discontinuation, with cessation of bisphosphonate treatment, residual effects can persist for several years after discontinuation,^66^^,^^67^ particularly for zoledronic acid.^67^ Therefore, when treatment targets have been met with a nonbisphosphonate, transitioning to a bisphosphonate may serve to maintain BMD gains. In one study of women with low bone mass, a single infusion of zoledronic acid was able to maintain BMD for up to 5 yr.^68^ Administration of periodic zoledronic acid, with a dose interval beyond 1 yr, might therefore be useful for long-term maintenance of treatment targets achieved. Periodic administration of oral bisphosphonates might also permit maintenance of BMD targets. For patients who have achieved treatment targets with bisphosphonate therapy, a pause in treatment (“bisphosphonate holiday”) may be considered. More information is needed to clarify the optimal dosing regimen to avoid rare but important side effects associated with bisphosphonate duration, such as atypical femur fractures and osteonecrosis of the jaw, while maximizing the likelihood of maintaining BMD.

### Limitations

The evidence used here is based almost solely on women self-reporting as White, primarily 60 yr of age and older. It needs to be clarified how recommendations should differ in lower risk populations (younger patients; men; women of other ethnicities, race, or geographical location). Furthermore, specific BMD levels are associated with a wide variation in absolute fracture risk, depending on ethnicity and geography. Indications for treatment vary in different regions of the world and the treatment targets suggested here should be modified according to local considerations. The treatment targets might also differ in patients with secondary osteoporosis conditions such as glucocorticoid-induced osteoporosis or in patients with diabetes mellitus.

A consensus is needed to confirm which fractures constitute a risk high enough to warrant initial use of osteoanabolic therapy. This is true for both patients with recent fractures and fractures that occurred more than 2 yr earlier. Consensus is also needed on defining an acceptable level of fracture risk after treatment. Reducing fracture risk is the treatment goal; currently, BMD targets are the best way to determine if this goal has been achieved. Determining whether BMD treatment targets have been met requires good quality, reliable BMD data, and interpretation. With a very low starting T-score, especially in the TH, treatment targets might not be attainable even with the most potent currently available therapy; however, the concept of choosing treatment sequences likely to attain BMD levels close to the target is still appropriate. The data to derive probabilities of attaining treatment targets are from post-hoc analyses, with two of the studies published only as abstracts at the current time. There is a paucity of evidence about treatment targets for patients who have fractures with starting T-scores above −2.5. Making treatment decisions based on at least a 50% probability of achieving the BMD target is intended as a general guide, not a rule.

The approach presented in this position statement does not take into account limitations imposed by health systems and insurers, cost, cost-effectiveness, or patient preferences. Osteoanabolic agents are relatively expensive (vs. antiresorptive agents) and often require a pre-approval process. Medication authorization, insurance coverage, medication cost, and out of pocket costs to consumers vary considerably around the world. These factors must be considered when treatment decisions are discussed with individual patients. There are few data addressing the optimal timing and effectiveness of repeat courses of osteoanabolic treatment after antiresorptive medication. Furthermore, this is not feasible in many countries.

Evidence about what to do once treatment targets are reached is also limited. While intravenous zoledronic acid may be the best option for many patients, the minimal effective dose and dosing interval to maintain BMD and fracture risk reduction, while avoiding rare complications, such as atypical femur fracture, is likely to vary among individuals and by prior osteoporosis treatment administered. In addition, some patients might not be candidates for bisphosphonates; these patients have very limited BMD maintenance options. Here, bisphosphonates are considered as a class with few distinctions among the agents, although most of the data available utilize alendronate or zoledronic acid.

Finally, including patients in decision-making regarding treatment targets, initial treatment selection and maintenance of treatment targets will likely add to the success of the goal directed therapy approach.^69^^,^^70^

### Conclusion

The ASBMR/BHOF Task Force on Goal-Directed Osteoporosis Treatment suggests that rapid fracture risk reduction should be the predominant treatment goal for patients at very high and imminent risk of fracture. The Task Force also recommends considering using BMD goals to guide clinical decisions for initiating and continuing treatment. Specific BMD targets and treatment decisions should be tailored to each patient’s clinical risk profile and BMD. Considerations presented here may be modified in the future as more evidence becomes available.
